# Maturity Classification of Rapeseed Using Hyperspectral Image Combined with Machine Learning

**DOI:** 10.34133/plantphenomics.0139

**Published:** 2024-03-26

**Authors:** Hui Feng, Yongqi Chen, Jingyan Song, Bingjie Lu, Caixia Shu, Jiajun Qiao, Yitao Liao, Wanneng Yang

**Affiliations:** ^1^ National Key Laboratory of Crop Genetic Improvement, National Center of Plant Gene Research (Wuhan), Hubei Hongshan Laboratory, Huazhong Agricultural University, Wuhan, 430070 Hubei, PR China.; ^2^ Shenzhen Institute of Nutrition and Health, Huazhong Agricultural University, Wuhan, 430070 Hubei, PR China.; ^3^ College of Engineering, Huazhong Agricultural University, Wuhan, 430070 Hubei, PR China.

## Abstract

Oilseed rape is an important oilseed crop planted worldwide. Maturity classification plays a crucial role in enhancing yield and expediting breeding research. Conventional methods of maturity classification are laborious and destructive in nature. In this study, a nondestructive classification model was established on the basis of hyperspectral imaging combined with machine learning algorithms. Initially, hyperspectral images were captured for 3 distinct ripeness stages of rapeseed, and raw spectral data were extracted from the hyperspectral images. The raw spectral data underwent preprocessing using 5 pretreatment methods, namely, Savitzky–Golay, first derivative, second derivative (D2nd), standard normal variate, and detrend, as well as various combinations of these methods. Subsequently, the feature wavelengths were extracted from the processed spectra using competitive adaptive reweighted sampling, successive projection algorithm (SPA), iterative spatial shrinkage of interval variables (IVISSA), and their combination algorithms, respectively. The classification models were constructed using the following algorithms: extreme learning machine, *k*-nearest neighbor, random forest, partial least-squares discriminant analysis, and support vector machine (SVM) algorithms, applied separately to the full wavelength and the feature wavelengths. A comparative analysis was conducted to evaluate the performance of diverse preprocessing methods, feature wavelength selection algorithms, and classification models, and the results showed that the model based on preprocessing-feature wavelength selection-machine learning could effectively predict the maturity of rapeseed. The D2nd-IVISSA-SPA-SVM model exhibited the highest modeling performance, attaining an accuracy rate of 97.86%. The findings suggest that rapeseed maturity can be rapidly and nondestructively ascertained through hyperspectral imaging.

## Introduction

Oilseed rape is a major oilseed crop globally, which has an increase yield in recent years [1]. Owing to its substantial value in both the food and nonfood sectors, the demand for rapeseed has been steadily increasing [2]. Optimal seed maturity is paramount for successful breeding and maximizing yield potential. Premature or delayed harvesting may lead to diminished seed quality and decreased yield. It is important to note that vegetable oil production is mainly synthesized during the mature stage of seed development [3]. Nevertheless, simultaneous harvesting of rapeseed does not ensure uniform maturity due to the asynchronous flowering and pod dehiscence of sequential racemes [4]. Considering the aforementioned analysis, the classification of rapeseed maturity holds immense significance in enhancing the quality of rapeseed oil and expediting breeding research.

There has been considerable research conducted on the classification of crop maturity. Traditional analytical methods such as gas and high-performance liquid chromatography (HPLC), as well as chemical analysis methods like Kjeldahl and Soxhlet, have been used to assess crop maturity [5]. For instance, mangoes at different stages of maturity were harvested, preserved, and evaluated using an electronic nose or gas chromatography, followed by classification using multivariate statistics [6]. Similarly, the surface color and lycopene content of tomatoes at 7 different maturity stages were measured using a Minolta chroma meter and HPLC, enabling the study of the relationship between maturity, color, and lycopene content [7].

However, traditional methods may cause physical impacts on samples during the classification process, making them unsuitable for high-throughput analysis. As a result, other researchers have attempted to establish maturity prediction models based on measured physical indices. Artificial neural networks have been used to assess the maturity of oil palm fresh fruit bunches, relying on color and texture as key parameters [8]. In another study, 2 nondestructive parameters, namely, color and quality, were measured during the developmental and ripening stages, and a model was calibrated using a continuous dataset to classify tomato fruits at different maturity stages [9]. However, because of the black–brown color and small seed diameter of rapeseed during purchase and processing, achieving high accuracy in maturity classification based solely on physical indices is challenging.

Hyperspectral imaging (HSI) is a technology based on multiband image, which can simultaneously provide spatial and spectral information related to plant and biochemistry [10,11]. Compared to destructive sampling methods, it provides a time-saving and cost-effective approach [12,13]. Its applications include detecting protein content in rice [14], predicting soluble solids in apples [15], and predicting chlorophyll content in rapeseed [16]. HSI was also utilized for assessing crop maturity due to its high-throughput and nondestructive nature. For instance, in the case of maize, the average spectra of the embryo side, endosperm side, and both sides were extracted from hyperspectral images, and a robust model incorporating the partial least-squares discriminant analysis (PLS-DA) algorithm and feature wavelengths was developed to accurately evaluate maize seed maturity [17]. Similarly, hyperspectral data from different maturity stages of peanut samples were used to establish a discrimination model based on feature wavelengths using support vector machine (SVM), enabling the prediction of peanut cluster maturity [18]. The maturity of okra was estimated using an HSI system within the 400- to 1,000-nm wavelength range, and a maturity classification model incorporating effective wavelengths, texture features, and fused data was created [19]. By selecting feature wavelengths, the impact of nonlinearity can be reduced, resulting in more efficient data processing and increased accuracy and robustness of the model [20]. Consequently, it is common practice to establish maturity classification models by selecting feature wavelengths when using HSI to predict crop maturity. In a study on Camellia fruit, a classification model was developed using PLS-DA, and feature wavelengths were selected through principal component loadings, 2-dimensional correlation spectroscopy, and the uninformative variable elimination and successive projection algorithm (SPA) [21]. Another study utilized stability competitive adaptive reweighted sampling (CARS) to extract feature wavelengths from hyperspectral apple data and applied partial least-squares regression to predict soluble solid content and starch pattern index, enabling spatial distribution analysis of apple maturity [22]. For strawberry samples collected at early and mature stages, hyperspectral data were processed using sequential feature selection to select feature wavelengths, and a convolutional neural networks were used to classify spatial feature images corresponding to the strawberry samples’ feature bands, achieving an impressive 98.6% accuracy on the test set [23]. HSI technology, with its high-throughput capabilities, minimizes sample damage during measurement, eliminates chemical pollution, and enables rapid and efficient determination of sample maturity once the model is established. Therefore, it can be seen that the aforementioned study used a combination of HSI technology and commonly used classification algorithms to predict the maturity of crops, presenting a more effective approach for classifying rapeseed maturity.

## Materials and Methods

### Experimental and technical design

**Fig. 1.**
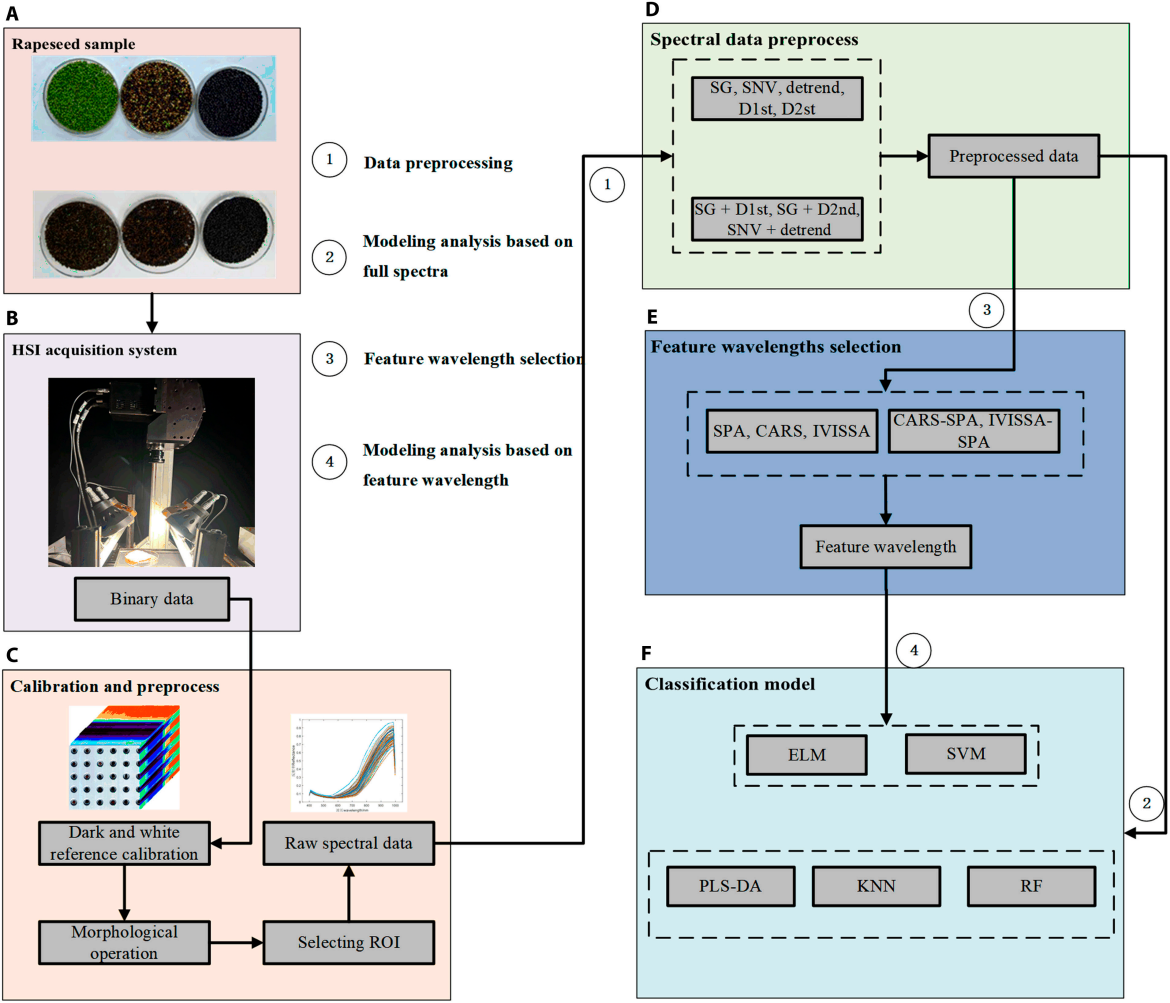
The overall process of rapeseed maturity classification. (A) Rapeseed at 3 different maturity levels. (B) HSI acquisition system. (C) Calibration and preprocess. (D) Spectral data preprocess. (E) Feature wavelengths selection. (F) Classification model.

This study can be summarized as follows: collecting spectral images of rapeseed at various maturity stages and extracting spectral data from the regions of interest. Using multiple preprocessing techniques to enhance the signal-to-noise ratio and using diverse classification algorithms to establish a comprehensive maturity classification model. Selecting feature wavelengths using multiple feature wavelength selection algorithms, constructing a classification model based on these feature wavelengths, comparing the performance of various wavelength selection algorithms and modeling algorithms, and identifying the optimal model for predicting rapeseed maturity. The key steps involved in the systematic classification of rapeseed maturity using HSI are illustrated (Fig. 1). The primary script and dataset used during the experimental procedure are accessible via the following link: http://plantphenomics.hzau.edu.cn/usercrop/Rice/download. The “reflectance.xlsx” file in the folder contains spectral reflectance data. Different sheet names correspond to the reflectance data obtained after respective preprocessing. The “code” folder contains the code for classification and feature wavelength extraction.

### Materials

The high-quality rapeseed variety “Huayouza 62” was utilized as a sample for this experiment. This variety holds a certain level of representativeness in the Chinese rapeseed industry, making the research results more applicable and generalizable. The seeds were sown on 20 October 2020, in the experimental field of Huazhong Agricultural University in Wuhan, China. From 19 April 2021 to 17 May 2021, a total of 7 batches of experimental samples were collected. The green maturity stage was collected on 19 and 23 April, the yellow maturity stage was collected on 28 April and 2 May, and the fully mature stage was collected on 7, 12, and 17 May. According to the sampling time and color of the siliques, they were divided into 3 categories. The green maturity stage is characterized by green color, the yellow maturity stage exhibits a mixture of reddish-brown and yellow rapeseeds, and the fully mature stage tends to have a black color. Ten random sampling points in the filed were selected for each batch, and a unit area of 1 m × 1 m was used. At each sampling point, 5 rapeseed plants were harvested by collecting the pods located at the bottom of the main plant. Then, the collected pods were peeled off manually in the laboratory to obtain rapeseed seeds. The rapeseed pods were stored in a properly ventilated environment, with a temperature between 20 and 25 °C. During this process, the water content of the seeds is gradually reduced to a uniform level, and with the drying process, the green and yellow seeds will naturally turn black (Fig. 1A). The experiment resulted in a total of 1,500 rapeseeds of varying maturity levels, including 400 seeds in the green maturity stage, 400 seeds in the yellow maturity stage, and 700 seeds in the fully mature stage.

### Hyperspectral acquisition system

The hyperspectral images of rapeseed samples were obtained by the system (Fig. 1B), which includes a hyperspectral camera (with a spectral range of 400 to 1,000 nm, a slit width of 25 μm, a spectral resolution of 1.9 nm, and a total of 314 bands), a halogen lamp (with a power of 150 W), an electrically driven moving carrier platform (with a moving speed of 2 mm/s), and a computer. Each sample obtained after shooting was saved in binary data stream format with a size of 3.16 gigabytes.

Hyperspectral images are susceptible to uneven illumination and dark current during the acquisition process [24]. Therefore, the system was preheated for 20 min prior to capturing the images, and Eq. 1 was used for calibration. In the formula, *I*_c_ represents the calibrated image, *I*_r_ denotes the original captured image, *I*_d_ refers to the dark reference image obtained with all light sources blocked, and *I*_w_ represents the white reference image captured using polytetrafluoroethylene.

$$I_{c}=\frac{I_{r}-I_{d}}{I_{w}-I_{d}}*100\%$$ (1)

### Hyperspectral image acquisition and preprocessing

**Fig. 2.**
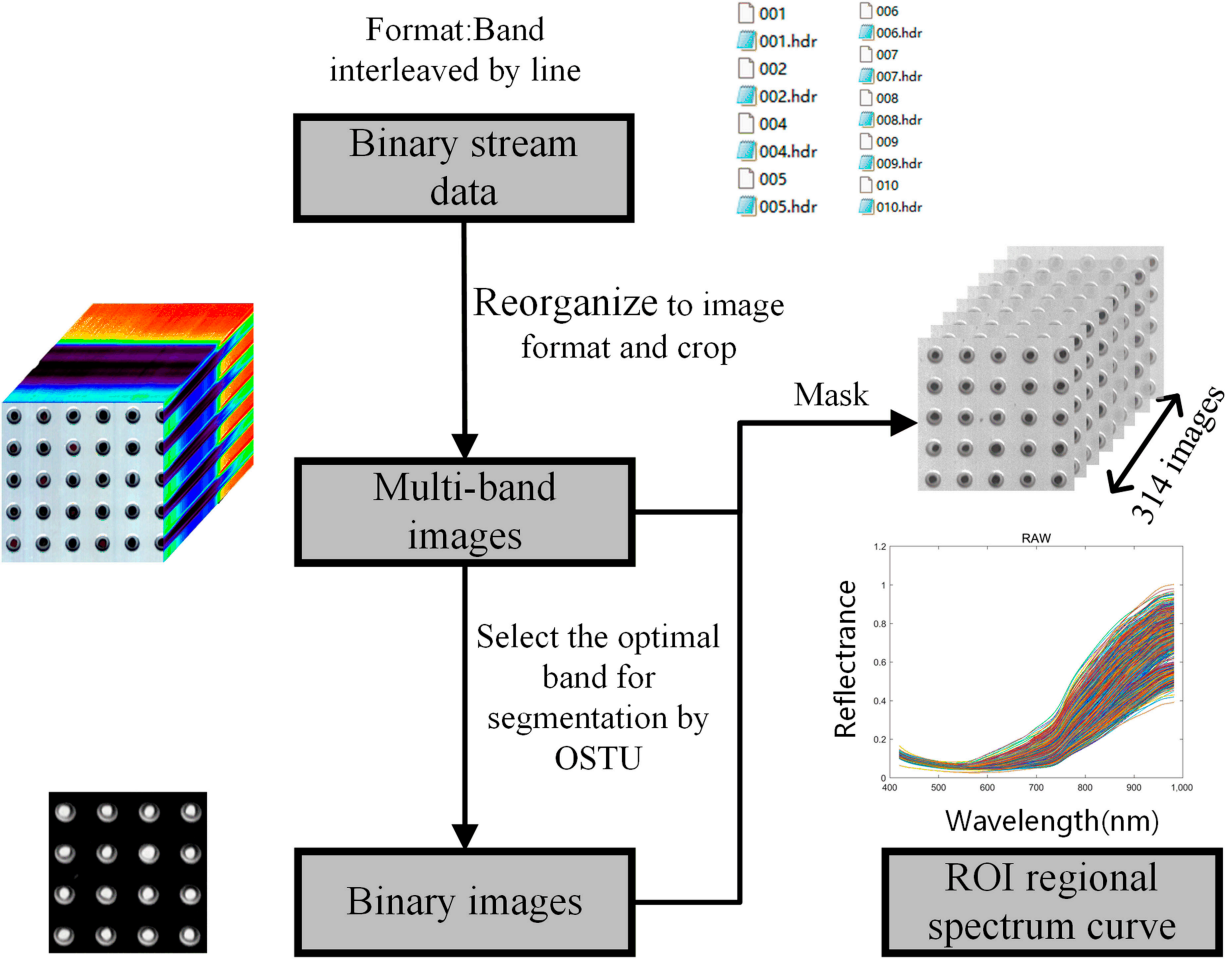
Spectral data processing procedures.

Figure 2 illustrates the complete processing flow for extracting spectral reflectance from binary value data. Full-band spectral images of each sample were obtained by reorganizing the binary data streams obtained from the HSI system. The hyperspectral images were cropped using ENVI 5.1 software (Environment for Visualizing Images software, Research Systems Inc., Boulder, CO, USA) to remove unnecessary regions and improve processing speed. The OTSU algorithm [25] was used to segment the image at 440-nm wavelength (which had the best contrast between the background plate and rapeseed spectrum), resulting in a binary image of rapeseed. The connected domains were labeled to extract the region of interest from the rapeseed images, and the full-band reflectance curve was obtained using the binary image mask of the full-band image.

Various preprocessing operations were applied to mitigate the influences of sample variations, light scattering, and baseline drift [26]. The preprocessing algorithms included Savitzky–Golay (SG), first derivative (D1st), second derivative (D2nd), standard normal variate (SNV), and detrend algorithms. In addition, considering their specific characteristics, SG was combined with D1st, SG was combined with D2nd, and SNV was combined with detrend to preprocess the raw spectral data. Kennard–Stone algorithm [27] was used to divide the data into training set and testing sets in a 3:1 ratio (training set, 1,125; testing set, 375). In the present study, equal proportions of green, yellow, and fully ripe stages were ensured within both the training and testing datasets. The training dataset is used for model training, whereas the testing dataset is used as a means of assessing the classification performance of the model.

### Feature wavelength selection

Hyperspectral images encompass a substantial volume of spectral data, often exhibiting redundancy, necessitating the selection of pertinent feature wavelengths. The study used 3 distinct algorithms, namely, the SPA, CARS, and iterative spatial shrinkage of interval variables (IVISSA), for extracting feature wavelengths. Subsequently, a combination of 2 of these algorithms, based on their respective characteristics, was used to enhance the efficiency of feature wavelength selection.

SPA is a deterministic search technique known for its reproducible results and superior validation set selection capability [28]. The algorithm identifies the largest projected wavelength as the feature wavelength, thereby mitigating the collision effect of the original input. The experiment established a range of 10 to 100 wavelength variables, and the determination of feature wavelengths relied on the alteration in root mean square error (RMSE). CARS adopts the principle of “survival of the fittest” to select the feature wavelengths, using the wavelength combination with the smallest RMSE of cross-validation among the results of 5 runs [29]. In this study, Monte Carlo sampling was performed 1,000 times with the implementation of the “center” preprocessing method. To address the instability of CARS in selecting spectral features, the feature wavelengths that yielded the minimum RMSE of cross-validation across 5 separate runs were selected. IVISSA is an algorithm for selecting wavelength intervals based on the VISSA algorithm. It combines global and local search to optimize the position, width, and combination of spectral intervals iteratively [30].

CARS algorithm performs poorly in reducing the dimensionality of rapeseed spectral data but shows good modeling performance on the reduced data. Compared to using CARS directly, CARS-SPA reduces the number of variables and provides more information [31]. While IVISSA shows good performance in selecting feature wavelengths, there is still spectral overlap. To mitigate the issue of data collinearity, the use of SPA is recommended. Recent studies have reported positive results using IVISSA-SPA for secondary extraction of spectral data [32,33].

### Classification model

In this section, 5 classification algorithms were compared, including extreme learning machine (ELM), *k*-nearest neighbor (KNN), random forest (RF), PLS-DA, and SVM, to establish the best model for classifying rapeseed at different maturity stages.

ELM is an enhanced one-way feedback neural network algorithm that is based on a feedforward neural network [34]. When utilizing ELM to construct the discriminative model for rapeseed maturity, the activation function used is the sigmoid function. The number of neurons in the ELM is set within the range of 30 to 100, with a step size of 10, while adjusting the number of neurons in the hidden layer to obtain the optimal configuration for various spectral data.

KNN algorithm is a frequently used supervised learning method that exhibits good classification performance while requiring relatively few parameters [35,36]. When utilizing KNN for classification, the selection of the *K* value notably affects the classification accuracy. In general, as the *K* value increases, the probability of accurate classification also rises. The *K* values in the model range from 5 to a maximum of 30, incrementing by a step size of 1.

RF is a decision tree ensemble model that utilizes Bagging as a framework [37]. RF uses an ensemble approach where a substantial number of decision trees are generated, with each tree being trained on the original training data. The output class is determined through majority voting among the trees [38]. In this study, the parameter “*N*” value of the Bagging framework is configured as 500, the maximum depth of the decision tree “*M*” ranges from 1 to 20, with a step size of 1 for the grid search.

The PLS-DA algorithm, a supervised classification method based on partial least-squares regression analysis, incorporates dimension reduction by combining predictors to generate latent variables that exhibit the highest correlation with the targeted outcomes [39,40]. The PLS-DA method finds wide application in diverse fields, including chemical analysis [41]. The parameter “*N*” in the PLS-DA model holds significant importance. If set too small, the discriminant model becomes inaccurate because of insufficient information. Conversely, setting the value too large increases the likelihood of overfitting.

SVM is a classical supervised machine learning model capable of classifying both linear and nonlinear models, finding widespread application across various fields. For this study, the SVM algorithm utilizes the radial basis function as its kernel function, and the penalty factor “*C*” and kernel parameter “*G*” undergo optimization through the 5-fold cross-validation method and the particle swarm optimization algorithm. “*C*” represents the penalty coefficient of the objective function, and its range is set from 0 to 100.

### Model evaluation

The extraction of rapeseed reflectance was conducted in this study using ENVI v5.3, while preprocessing, feature wavelength extraction, and modeling tasks were accomplished using MATLAB 2019a.

Accuracy is applied as the evaluation metric to assess the predictive performance of the model in identifying rapeseed maturity. In Eq. 2, true positive (TP) denotes correctly classified positive samples, true negative (TN) denotes correctly classified negative samples, false positive (FP) represents incorrectly classified positive samples, and false negative (FN) represents incorrectly classified negative samples.

$$\text{Accuracy}=\frac{\text{TP}+\text{TN}}{\text{TP}+\text{FN}+\text{TN}+\text{FP}}$$ (2)

## Results

### Spectrum characteristics analyses

**Fig. 3.**
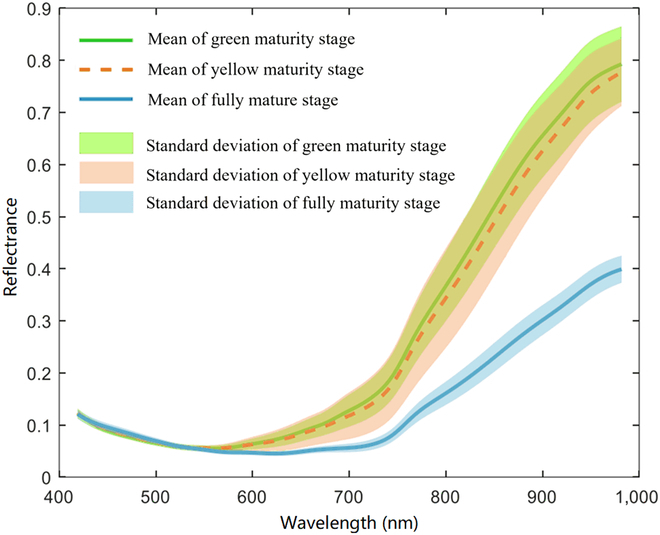
Mean and standard deviation of rapeseed spectral reflectance.

**Fig. 4.**
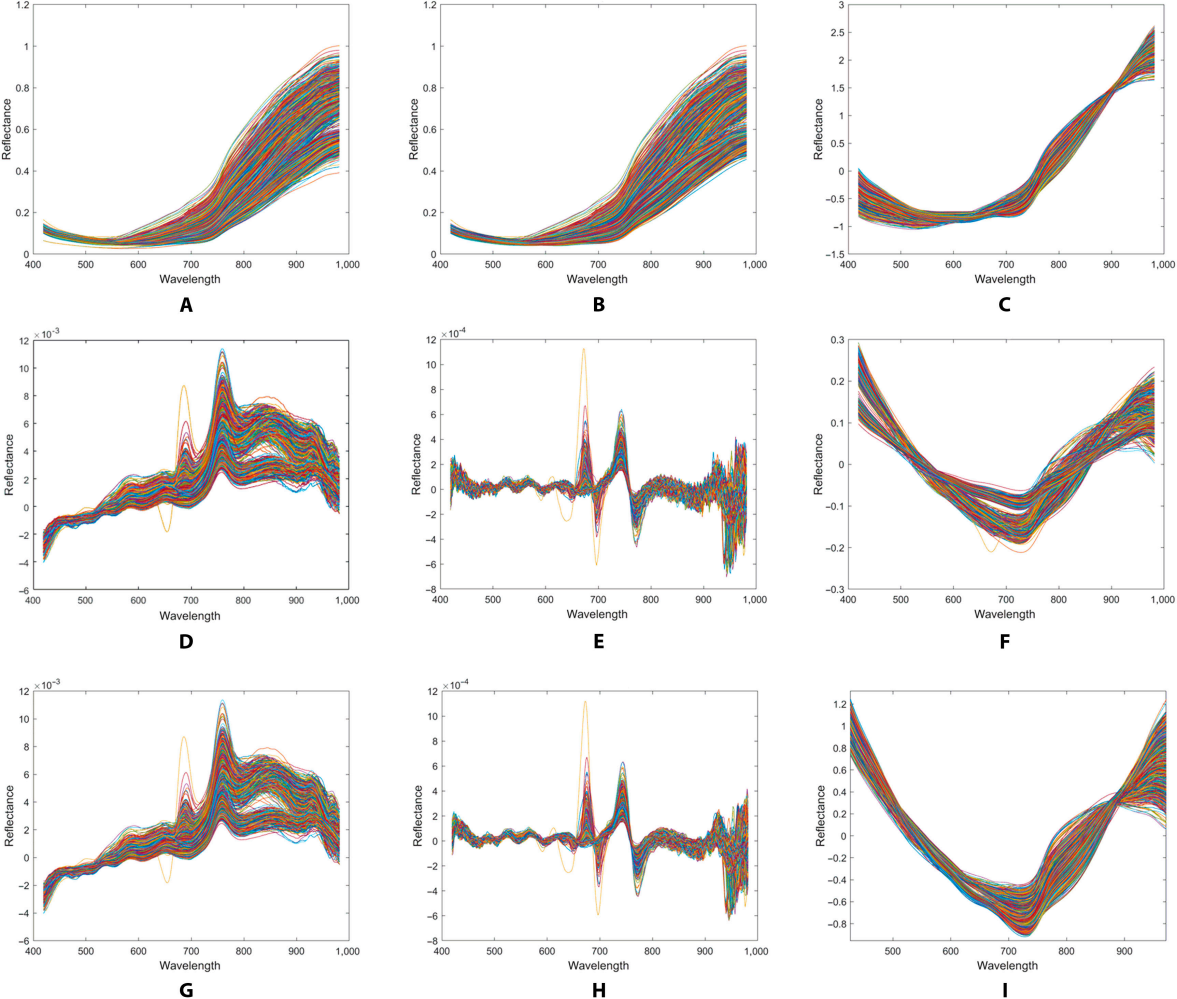
Spectral curves of different preprocessing methods. (A) Raw. (B) SG. (C) SNV. (D) D1st. (E) D2nd. (F) Detrend. (G) SG + D1st. (H) SG + D2nd. (I) SNV + detrend.

The average spectral curves were depicted in Fig. 3 reveal the similarity in spectral curves among the 3 maturity levels of rapeseed between 420 and 530 nm. Within the 530- to 720-nm range, the reflectance of the green and yellow maturity stages exhibits similarity, with a slight distinction observed between the fully mature stage and the other 2 stages. Within the 720- to 982-nm range, the disparity between the mature stage and the other 2 stages progressively increases. Figure 4A shows the raw reflectance curves of rapeseed with different maturity levels. The experiment was restricted to a wavelength range between 420 and 982 nm due to the substantial noise observed in the spectra between 400 to 420 nm and 982 to 1,000 nm.

### Spectral data preprocessing results

The spectral curves processed with different methods show significant differences (Fig. 4). Applying SG processing to the original spectra reduces the fluctuations in the spectral curve, resulting in a smoother appearance (Fig. 4B). Following SNV processing, the spectral changes increase, while the curve exhibits a relatively consistent trend (Fig. 4C). Derivative processing results in inconsistent changes in the spectral curves of rapeseeds at different maturity levels, with relatively large fluctuations in the latter half of the spectrum (Fig. 4D and E). Applying detrend processing to the spectra results in differing spectral trends among rapeseeds at different maturity levels (Fig. 4F and G). The application of SG + derivative preprocessing to the spectra reveals a reduction in fluctuations within the 800- to 1,000-nm wavelength region. The application of SNV + detrend preprocessing amplifies the curve’s trend while reducing the areas of spectral discrepancy (Fig. 4I).

### Modeling analysis based on full wavelengths

The modeling results including accuracy and precision from the entire wavelength (Table 1 and Table S10) revealed substantial variations when using different preprocessed spectral data as input. Among the 5 classification algorithms, using preprocessed spectral data with D1st, SG + D1st, and SG + D2nd yields superior modeling outcomes compared to using the original spectral data. Except for the RF model, D2nd outperforms the original spectral model in all models, and SNV + detrend surpasses the original spectral model in all models except for the KNN model. Overall, D1st, D2nd, SG + D1st, SG + D2nd, and SNV + detrend contribute to enhancing the signal-to-noise ratio of rapeseed spectral data, as indicated by the accuracy results.

**Table 1.** Accuracy of a full wavelength classification model

**Preprocessing**	**Training set accuracy (%)**					**Test set accuracy (%)**				
	**ELM**	**KNN**	**RF**	**PLS-DA**	**SVM**	**ELM**	**KNN**	**RF**	**PLS-DA**	**SVM**
Raw	92.27	91.20	100	85.97	93.60	92.00	89.16	91.2	80.51	92.36
SG	92.27	93.6	100	91.94	93.60	92.00	86.49	92.44	88.29	93.16
D1st	97.60	95.47	100	93.91	98.49	97.33	89.16	93.87	90.07	97.60
D2nd	97.60	93.87	98.31	93.73	98.49	96.00	91.73	93.87	87.60	97.87
SNV	91.73	91.20	95.91	88.89	95.73	90.67	88.98	93.60	85.96	91.02
Detrend	91.73	91.20	96	91.86	95.47	91.29	88.44	90.67	90.50	90.84
SG + D1st	98.31	95.73	100	92.00	98.76	97.33	89.42	93.87	90.04	97.60
SG + D2nd	98.22	95.73	100	93.67	98.49	97.33	89.33	93.87	88.30	97.07
SNV + detrend	96.53	95.2	100	89.02	95.47	96.17	89.51	94.13	85.76	93.16

Among the 5 modeling methods, ELM and SVM exhibited the highest modeling performance, with an overall prediction accuracy exceeding 92%. SG + D1st yields the highest modeling result for ELM, achieving a prediction accuracy of 97.33%. D2nd achieves the best modeling result for SVM, with a prediction accuracy of 97.87%. Tables S1 to S3 present the classification precision and recall for each category, thereby offering an in-depth insight into the model’s performance across various categories and enabling a comprehensive evaluation of its overall performance. The precision rates of the 3 categories show that the evaluation metric for the third category is noticeably higher than that of the first 2 categories. This aligns with the consistent trend in the average spectral curves of the 3 categories. Therefore, the judgment for the first 2 categories is more critical. In modeling with the full wavelength, ELM and SVM models demonstrate better capability in distinguishing between the green ripening period and the yellow ripening period compared to other models.

### Feature wavelength selection results

The results demonstrate that applying D1st, D2nd, SG + D1st, SG + D2nd, and SNV + detrend enhances the accuracy of the rapeseed maturity model (Table 1). Consequently, feature wavelengths were extracted from the raw spectral data, and the data underwent preprocessing using these 5 methods.

**Fig. 5.**
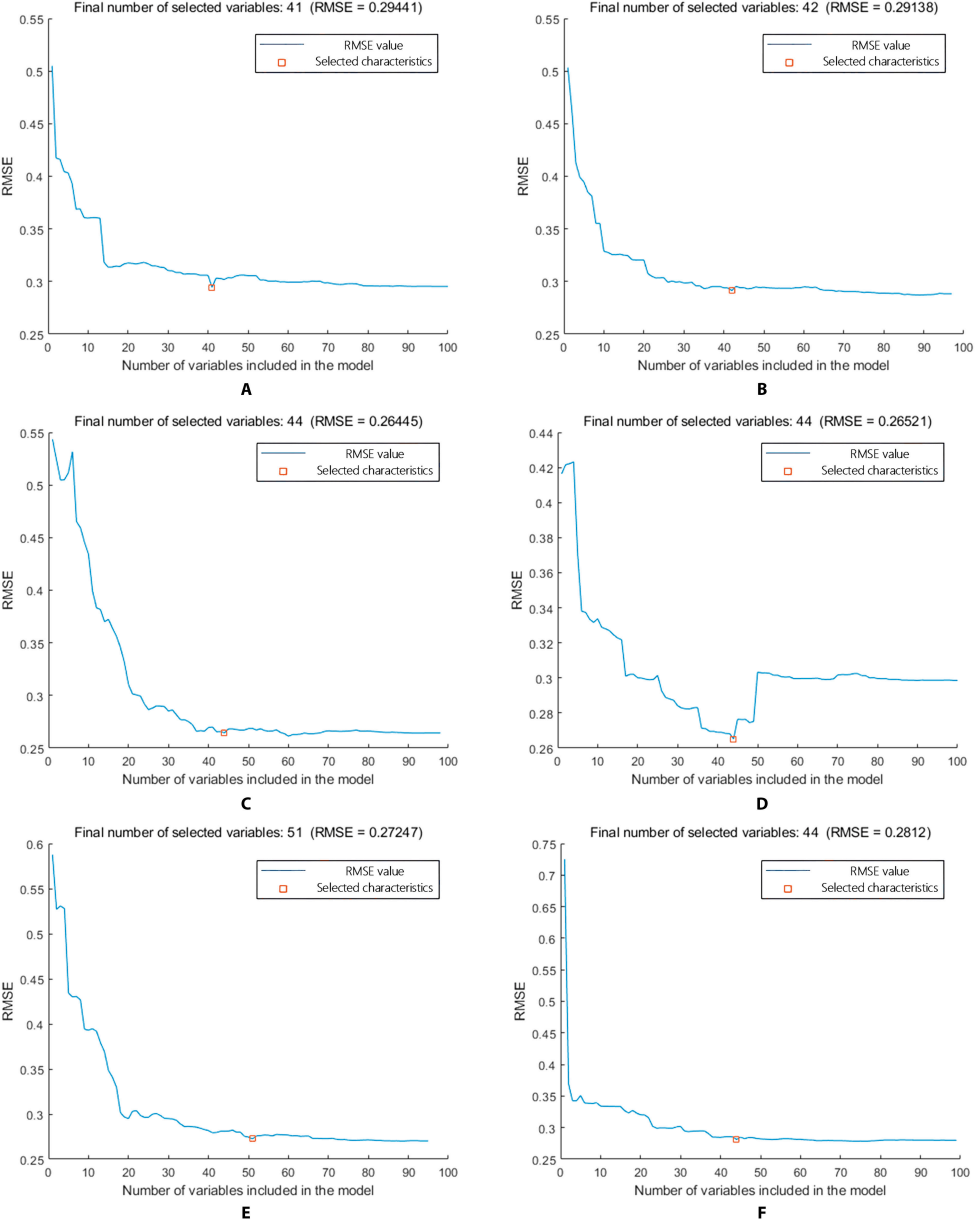
RMSE curve obtained using the SPA algorithm on the source data and preprocessed data. (A) Raw. (B) D1st. (C) D2nd. (D) SG + D1st. (E) SG + D2nd. (F) SNV + detrend.

**Fig. 6.**
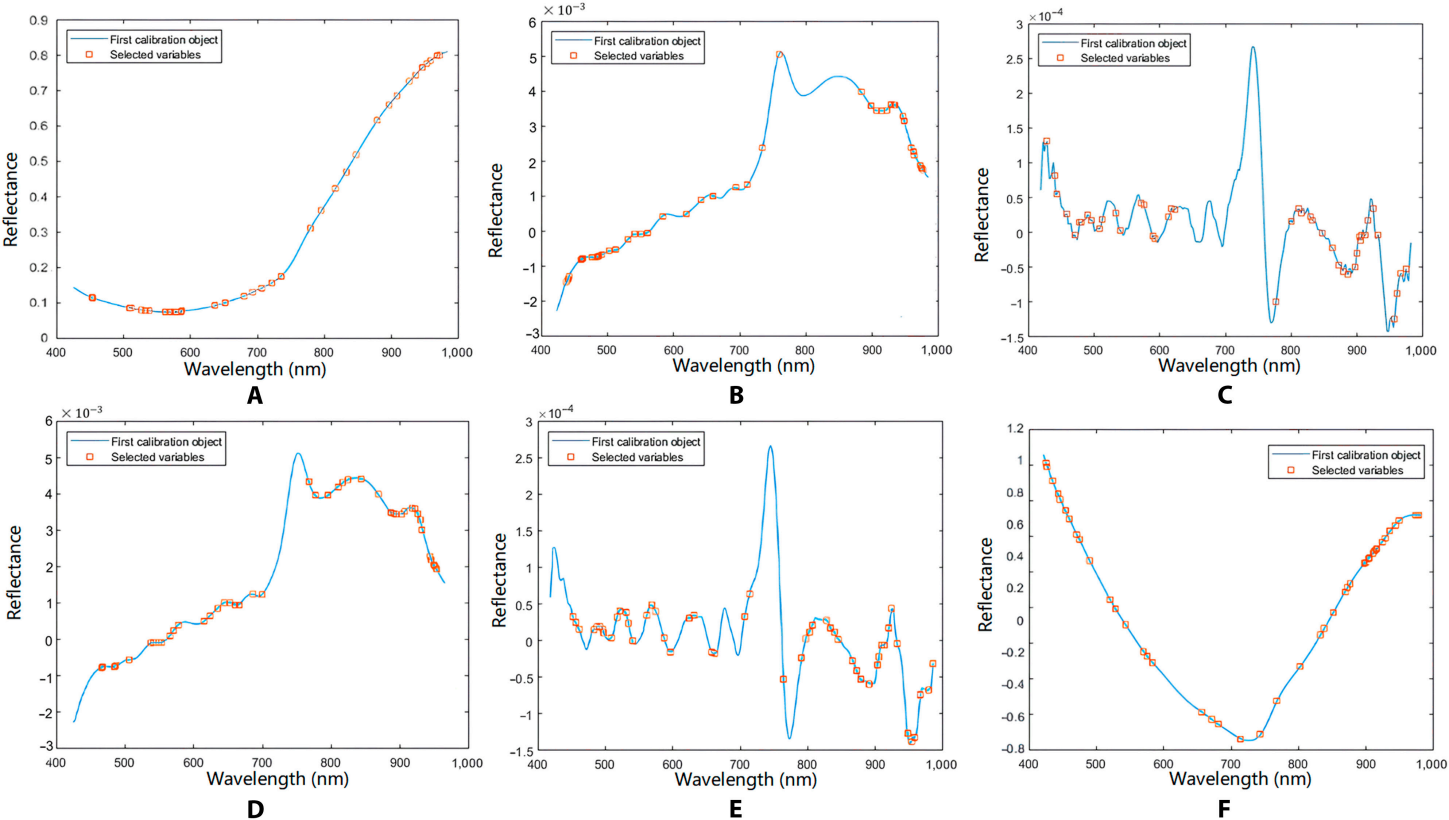
The distribution of feature wavelengths selected from the raw data and preprocessed data using the SPA algorithm. (A) Raw. (B) D1st. (C) D2nd. (D) SG + D1st. (E) SG + D2nd. (F) SNV + detrend.

The RMSE curves of different preprocessing algorithms using the SPA algorithm were obtained, taking SPA as an example (Fig. 5). The number of feature wavelengths obtained by the SPA algorithm on the raw data (Fig. 5A) and data processed by D1st (Fig. 5B), D2nd (Fig. 5C), SG + D1st (Fig. 5D), SG + D2nd (Fig. 5E), and SNV + detrend (Fig. 5F) are 41, 42, 44, 44, 51, and 44, respectively. The feature wavelengths selected from the raw spectral data are predominantly concentrated in the 450- to 550-nm band, with scattered distribution in other bands (Fig. 6A). D1st-selected feature wavelengths are mainly found in the 450- to 550-nm and 850- to 1,000-nm ranges, with scattered distributions in other bands, but primarily concentrated at the peaks of those bands’ wavelengths (Fig. 6B). The wavelengths within the range of 600 to 800 nm were not selected using D2nd, while other wavelengths exhibit scattered distribution, primarily situated at the turning points of various wavelength bands (Fig. 6C). Compared with D1st, the SG + D1st method selects a greater number of feature wavelengths in the 600- to 900-nm range, primarily concentrated at the local peak points and turning points of the wavelengths (Fig. 6D). The distribution of selected feature wavelengths using SG + D2nd is similar to that of D2nd (Fig. 6E). The feature wavelengths selected through the combination of SNV and detrend are predominantly concentrated around the 900-nm wavelength (Fig. 6F)

The results of applying 5 feature wavelength selection algorithms to spectral data using various preprocessing methods are presented in Table 2. This includes the number of obtained wavelengths and the corresponding ratio of feature wavelengths to the original spectral band (Table 3). The detailed distribution of the selected feature wavelengths can be found in Figs. S1 to S4.

**Table 2.** Number of feature wavelength selection algorithm extraction

**Preprocessing**	**Wavelength selection algorithm**				
	**SPA**	**CARS**	**IVISSA**	**CARS-SPA**	**IVISSA-SPA**
Raw	41	111	90	37	35
D1st	42	157	102	37	66
D2nd	44	157	123	36	70
SG + D1st	44	85	88	42	53
SG + D2nd	50	107	114	35	68
SNV + detrend	44	73	90	43	38

**Table 3.** The proportion of the feature wavelength to the full band

**Wavelength selection**	**Percent**
SPA	13.89%–17.29%
CARS	24.75%–53.22%
IVISSA	26.10%–41.69%
CARS-SPA	12%–14.57%
IVISSA-SPA	11.19%–23.73%

### Modeling analysis based on feature wavelength

On the basis of the above classification results, the ELM and SVM models demonstrated the highest classification accuracy, leading to their selection for establishing prediction models using the feature wavelengths. According to Tables 4 and 5 and Tables S11 and S12 in terms of wavelength selection algorithms, the overall accuracy ranking from highest to lowest is IVISSA-SPA, CARS, IVISSA, CARS-SPA, and SPA. The feature wavelengths extracted using the CARS and IVISSA-SPA algorithms exhibited superior prediction accuracy compared to the other 3 feature wavelength selection methods. D2nd-IVISSA-SPA-SVM achieved the highest accuracy among all the models, with an impressive accuracy rate of 97.86% and a precision rate reaching 94.98%. In terms of classification precision for individual categories, the green and yellow mature stage categories also demonstrated high classification performance (Tables S4 to S9).

**Table 4.** Feature wavelength classification model using ELM

**Preprocessing**	**Training set accuracy (%)**					**Test set accuracy (%)**				
	**SPA**	**CARS**	**IVISSA**	**CARS-SPA**	**IVISSA-SPA**	**SPA**	**CARS**	**IVISSA**	**CARS-SPA**	**IVISSA-SPA**
Raw	92.09	93.87	92.89	94.93	92.53	90.4	92.36	91.2	90.31	91.91
D1st	94.67	96.88	96.71	96.98	97.87	92.53	94.93	95.2	94.93	96
D2nd	95.2	98.13	97.51	94.84	97.51	94.76	97.6	96	93.87	96.53
SG + D1st	94.76	98.31	97.15	97.42	96	92.44	96.8	96.27	94.13	93.42
SG + D2nd	96.53	96.53	96.27	94.93	98.13	91.73	96	95.11	91.02	96
SNV + detrend	93.33	95.2	96.17	93.07	96.8	92.36	93.6	92.27	91.91	94.13

**Table 5.** Feature wavelength classification model using SVM

**Preprocessing**	**Training set accuracy (%)**					**Test set accuracy (%)**				
	**SPA**	**CARS**	**IVISSA**	**CARS-SPA**	**IVISSA-SPA**	**SPA**	**CARS**	**IVISSA**	**CARS-SPA**	**IVISSA-SPA**
Raw	92	93.87	93.87	93.6	93.6	89.87	93.6	93.33	91.02	91.73
D1st	94.4	97.51	97.07	97.07	96.53	91.91	96.27	96.62	95.47	94.13
D2nd	96.53	98.22	99.64	100	98.22	94.49	97.6	97.33	96	97.86
SG + D1st	94.93	97.96	97.07	96	95.91	93.16	97.6	95.47	95.47	95.73
SG + D2nd	94.13	96.27	97.6	96.09	98.4	92.71	95.47	96	95.2	97.07
SNV + detrend	93.69	94.13	94.93	95.2	95.2	93.07	93.87	93.16	92.8	92.18

While models based on feature wavelengths may sacrifice some spectral information, they successfully alleviate the redundancy present in the original data. Thus, selecting an appropriate feature wavelength method is crucial for establishing precise models. The accuracy of the preprocessed data surpasses that of the original spectra, signifying the effective enhancement of signal-to-noise ratio and robustness of the model through preprocessing. D2nd exhibited the highest average accuracy, demonstrating that derivative processing enhanced spectral sensitivity and effectively portrayed changes in the spectral curve contour, thereby accentuating the nuanced differences in spectral data across varying ripeness levels of rapeseed.

## Discussion

While several studies have explored the fusion of hyperspectral data and machine learning algorithms for nondestructive rapeseed quality parameter analysis, research concerning maturity detection in this domain remains limited. For example, some studies have used visible and near-infrared (NIR) hyperspectral data in conjunction with machine learning techniques to quantify nitrogen levels in rapeseed leaves [42]. Another study utilizes NIR HSI spectroscopy and chemometrics to assess the quality parameters of rapeseed [43]. This study initially verifies the viability of utilizing the full wavelength range for classifying rapeseed maturity. Subsequently, a series of feature selection algorithms is applied to extract feature wavelength from the full wavelength, with the goal of diminishing data redundancy while preserving classification precision. By analyzing the distribution of feature wavelengths extracted by each feature wavelength selection algorithm, most of them are located in the 700- to 900-nm interval range. Further, among the 5 feature wavelength extraction algorithms, the wavelengths extracted by CARS and IVISSA-SPA, which were the most effective in modeling, were located at the most 800 to 900 nm. Therefore, the wavelengths associated with rapeseed maturity may be located between 800 and 900 nm. This also corresponds to the average spectral curves at 3 different maturity stages of rapeseed in the “Spectrum characteristics analyses” section. In bands with greater differences in reflectance, it is easier to separate rapeseeds at different maturity levels. Another study that uses HSI to assess the ripeness of *Camellia oleifera* fruit similarly identified feature bands within the 800 to 900 range [21]. The ripeness of oil palm fruit is determined through a hyperspectral system, and it was found that the 750- to 900-nm wavelength range (NIR region) can accurately distinguish 3 different maturity categories, similar to the conclusion in this paper [44]. Interestingly, the spectral curves for the 3 maturity stages, including underripe, ripe, and overripe, are also consistent with the trends observed in the spectral curves in this study. Cellular structure and leaf water content contribute importantly to the 700- to 900-nm range of the NIR and short-wave infrared bands [45], when investigating the relationship between water content and spectral bands in pepper seeds, a similar identification was made around the 800- to 900-nm range (NIR) [46]. This further confirms that moisture is a crucial component influencing the maturity of rapeseeds. In the study utilizing HSI to predict lipid content in oilseed crops such as almonds, feature bands around the 900-nm range were also located [47]. Therefore, moisture and oil content may be crucial components influencing the spectral reflectance at different maturity stages for the assessment of rapeseed maturity. The spectrum processed by D2nd may better emphasize the spectral differences related to maturity-associated information, such as moisture content. IVISSA exhibits good performance in selecting feature wavelengths, but it tends to choose a relatively large number of feature wavelengths. The use of SPA can reduce redundancy and collinearity in the selected feature wavelengths. Their combination may result in a more accurate coverage of the selected wavelengths, specifically capturing key information related to the ripeness of rapeseeds. The prediction of rapeseed ripeness may involve complex nonlinear relationships. The combination of D2nd-IVISSA-SPA preprocessing and feature selection may make the input data more aligned with the classification characteristics of the SVM model.

In this research, there are potential influencing factors such as the impact of geographical location on the growth process of rapeseed. These variations might result in different spectral characteristics of rapeseeds under varying growth conditions. In further studies, validation across different geographical locations, years, and planting conditions will be considered to enhance the model’s generalizability. Simplified sensor technology and optimized data collection processes will be considered to achieve low-cost prediction of rapeseed ripeness in practical applications. Notwithstanding certain limitations in this investigation, such as the opportunity for refining rapeseed classification grades and harnessing additional spectral image information beyond reflectance for classification assistance, we anticipate that this research will streamline the implementation of rapeseed maturity classification models and offer substantial backing for rapeseed maturity grading.

The research confirms the potential of HSI technology for nondestructive detection of rapeseed maturity. Hyperspectral images of dried rapeseeds were obtained, and reflectance values from the region of interest were extracted. SG, D1st, D2nd, SNV, detrend, SG + D1st, SG + D2nd, and SNV + detrend were used to reduce the impact of noise generated during the imaging process. SPA, CARS, IVISSA, and their combination algorithms were applied for feature wavelength extraction, while ELM, KNN, RF, PLS-DA, and SVM were utilized to establish discrimination models.

A comparison was made among various preprocessing algorithms, feature wavelength extraction algorithms, and classification algorithms to assess their performance. The results demonstrate the effectiveness of the model that combines preprocessing, feature wavelength extraction, and machine learning algorithms in predicting the maturity of rapeseed grains. Among the models, the D2nd-IVISSA-SPA-SVM model exhibited the most favorable modeling effect. Importantly, the maturity classification model based on nondestructive and efficient HSI emerges as a promising tool for future rapeseed maturity classification. These findings offer valuable insights and inspiration for the advancement of innovative approaches in this field.

## Data Availability

All authors confirm that all raw experimental data are available upon request. The primary script and dataset used during the experimental procedure are accessible via the following link: http://plantphenomics.hzau.edu.cn/usercrop/Rice/download.
