# Supplementary material for: Maturity Classification of Rapeseed Using Hyperspectral Image Combined with Machine Learning
Source: Plant Phenomics. 2024 Mar 26;6:0139. doi: 10.34133/plantphenomics.0139 (PMC10976948; doi:10.34133/plantphenomics.0139)
Supplement: Supplementary 1 — Figs. S1 to S4 Tables S1 to S12 [file plantphenomics.0139.f1.pdf]

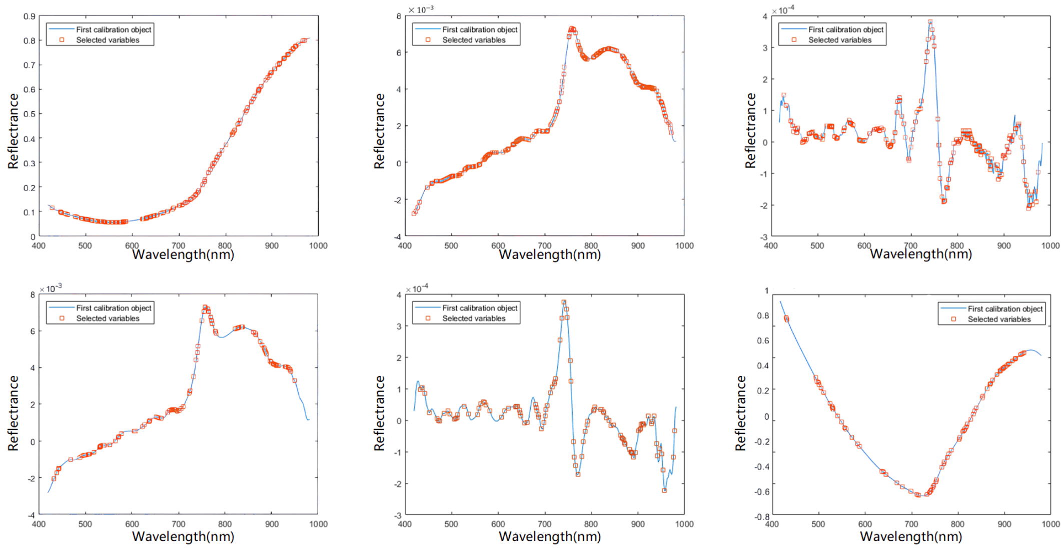

Fig. S1 Feature wavelengths obtained by using the CARS algorithm to screen the source data and preprocessed data; (**a**) Raw. (**b**) D1st. (**c**) D2nd. (**d**) SG+D1st. (**e**) SG+D2nd. (**f**) SNV+Detrend

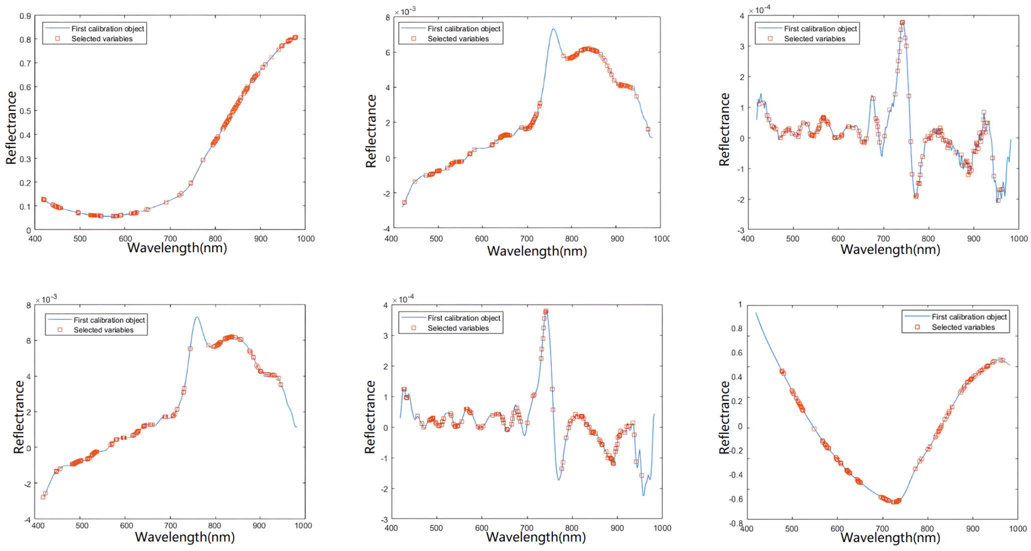

Fig. S2 Feature wavelengths obtained by using the IVISSA algorithm to screen the source data and preprocessed data; (**a**) Raw. (**b**) D1st. (**c**) D2nd. (**d**) SG+D1st. (**e**) SG+D2nd. (**f**) SNV+Detrend

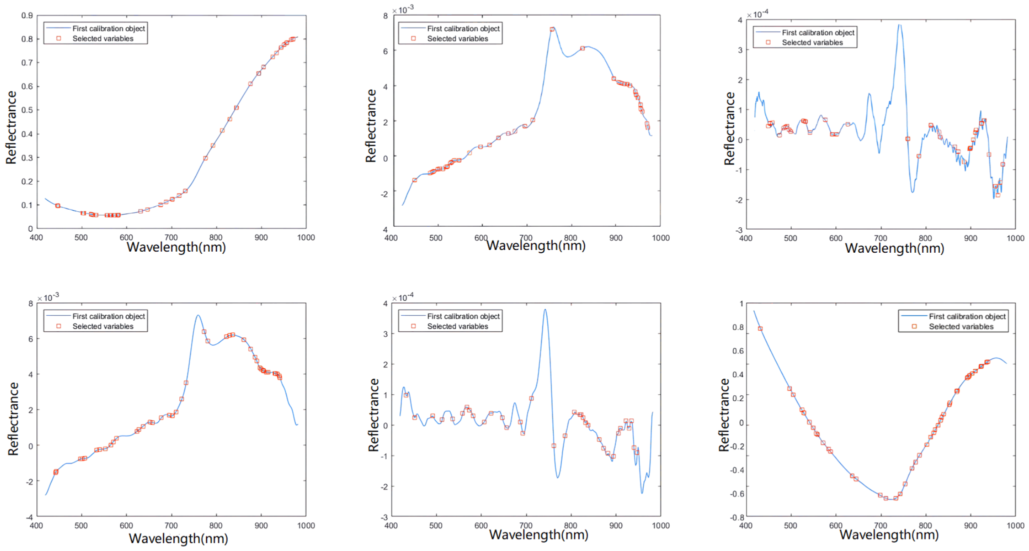

Fig. S3 Feature wavelengths obtained by using the CARS-SPA algorithm to screen the source data and preprocessed data; (**a**) Raw. (**b**) D1st. (**c**) D2nd. (**d**) SG+D1st. (**e**) SG+D2nd. (**f**) SNV+Detrend

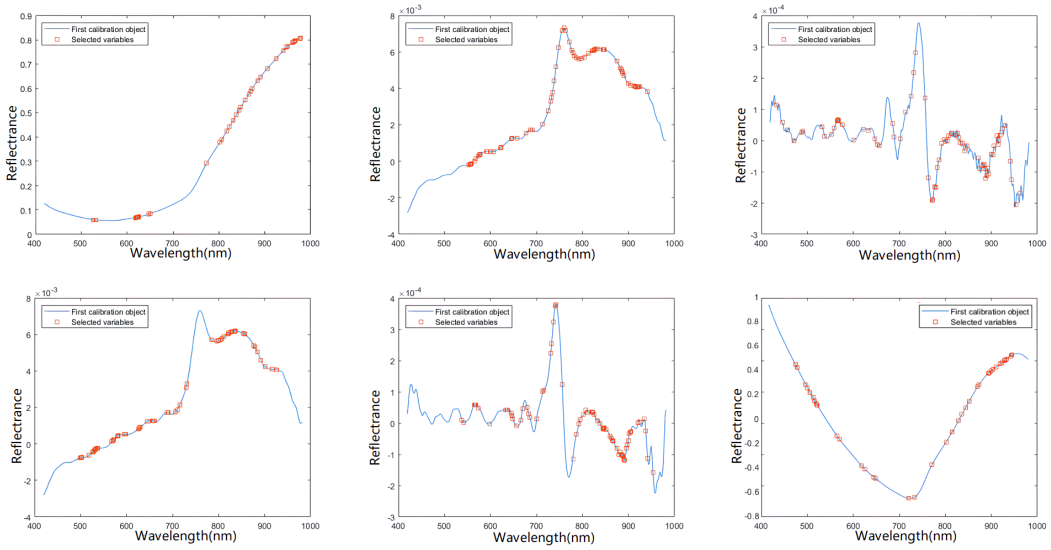

Fig. S4 Feature wavelengths obtained by using the IVISSA-SPA algorithm to screen the source data and preprocessed data; (**a**) Raw. (**b**) D1st. (**c**) D2nd. (**d**) SG+D1st. (**e**) SG+D2nd. (**f**) SNV+Detrend

Table S1 Precision and recall of full wavelength classification for the green mature stage

Preprocessing	precision (%)					recall (%)				
	**ELM**	**KNN**	**RF**	**PLS-DA**	**SVM**	**ELM**	**KNN**	**RF**	**PLS-DA**	**SVM**
RAW	96.61	85.64	86.32	69.44	87.85	75	100	83.67	64.94	96.61
SG	100	75.62	85.2 9	73.91	87.85	96.55	100	87.88	94.44	100
D1st	100	78.76	88.42	76.84	100	90	100	87.5	91.25	100
D2nd	100	82.56	90.2	80.22	100	92	**100**	88.46	96.05	100
SNV	100	84.7	79.44	83.16	100	89.16	100	86.73	98.75	100
Detrend	100	79.33	86.21	74.19	100	82	100	86.21	97.18	100
SG+D1st	97.67	79.17	89.25	76.84	100	**93.33**	100	86.46	**91.25**	97.67
SG+D2nd	100	79.17	89.32	73.91	100	88.89	100	88.46	94.44	100
SNV+Detrend	100	79.17	84.55	74.19	100	95	100	**94.9**	97.18	100

Table S2 Precision and recall of full wavelength classification for the yellow mature stage

Preprocessing	precision (%)					recall (%)				
	**ELM**	**KNN**	**RF**	**PLS-DA**	**SVM**	**ELM**	**KNN**	**RF**	**PLS-DA**	**SVM**
RAW	79.57	100	82.86	69.01	100	93.67	60.61	87	67.12	79.57
SG	95.0 8	100	87.8 8	91.8	100	75.32	52.3 5	84.4 7	65.12	95.0 8
D1st	92.31	100	88.35	88.73	91.26	100	61.76	89.22	74.12	92.31
D2nd	93	100	87.25	81.69	92.16	91	70.76	89.9	70.73	93
SNV	87.32	100	85.71	91.3	88.78	79.49	**58.18**	78	74.12	87.32
Detrend	78	100	88.89	86.36	88.78	88	**61.4**	88.89	63.33	78
SG+D1st	92.5	100	87.62	88.73	91.26	**97.37**	62.94	90.2	**74.12**	92.5
SG+D2nd	91.58	100	88	91.8	100	100	62.35	88.89	65.12	91.58
SNV+Detrend	95	100	94.32	86.36	88.78	91	62.94	**83**	63.33	95

Table S3 Precision and recall of full wavelength classification for the fully mature stage

Preprocessing	precision (%)					recall (%)				
	ELM	KNN	RF	PLS-DA	SVM	ELM	KNN	RF	PLS-DA	SVM
RAW	97.97	87.77	98.86	91.72	94.27	100	100	97.74	96	97.97
SG	87.74	89.68	99.43	95.92	94.27	100	100	100	99.3	87.74
D1st	98.67	92.05	100	100	100	100	100	100	99.26	98.67
D2nd	95	93.49	100	95.65	100	100	**100**	99.42	92.96	95
SNV	89.68	87.23	100	95.59	96.88	100	100	100	96.3	89.68
Detrend	94	90.82	100	93.62	96.88	97	100	100	94.96	94
SG+D1st	100	92.36	100	100	100	**100**	100	100	**99.26**	100
SG+D2nd	100	92.05	100	95.92	94.09	100	100	100	99.3	100
SNV+Detrend	95	92.36	100	93.62	96.88	100	100	100	94.96	95

Table S4 Precision and recall of the green mature stage category in feature wavelength modeling using ELM

Preprocessing	precision (%)					recall (%)				
	SPA	CARS	IVISSA	CARS-SPA	IVISSA-SPA	SPA	CARS	IVISSA	CARS-SPA	IVISSA-SPA
RAW	82.14	88.28	86.75	83.51	84.26	86.25	88.28	90	**91.01**	90.1
D1st	87.18	90.00	89.29	94.32	89.12	85	90.00	92.59	85.57	93.57
D2nd	86.62	95.06	95.89	93.41	95.83	89.13	**96.25**	87.5	83.33	91.09
SG+D1st	85.19	91.57	92.59	94.57	92.22	86.25	95	**93.75**	82.86	81.37
SG+D2nd	85.53	91.46	91.46	86.36	93.94	**81.25**	93.75	93.75	78.35	91.18
SNV+Detrend	88.31	86.62	84.34	84.69	87.04	85	87.86	87.5	85.57	**93.07**

Table S5 Precision and recall of the yellow mature stage category in feature wavelength modeling using ELM

Preprocessing	precision (%)					recall (%)				
	SPA	CARS	IVISSA	CARS-SPA	IVISSA-SPA	SPA	CARS	IVISSA	CARS-SPA	IVISSA-SPA
RAW	82.61	81.25	84.04	80.95	85.42	89.41	88.64	92.94	86.73	86.32
D1st	86.21	90.59	91.89	87.04	93.28	88.24	90.59	88.31	94.95	88.65
D2nd	88.81	96.43	89.13	82.11	92.17	86.23	**95.29**	96.47	92.86	96.36

SG+D1st	86.9	95.12	94.05	82.86	80.21	85.88	91.76	**92.94**	94.57	91.67
SG+D2nd	83.15	93.98	93.98	80.56	89.66	**87.06**	91.76	91.76	87.88	92.86
SNV+Detrend	85.39	87.77	87.80	85.71	92.05	89.41	86.52	84.71	84.85	**85.26**

Table S6 Precision and recall of the fully mature stage category in feature wavelength modeling using ELM

**Preprocessing**	**precision (%)**					**recall (%)**				
	**SPA**	**CARS**	**IVISSA**	**CARS-SPA**	**IVISSA-SPA**	**SPA**	**CARS**	**IVISSA**	**CARS-SPA**	**IVISSA-SPA**
RAW	100	99.21	99.19	99.42	100	91.85	94.72	90.37	**91.49**	95.53
D1st	100	100	100	100	100	100	100	100	100	100
D2nd	100	100	100	100	100	100	**100**	100	100	100
SG+D1st	100	100	100	100	100	100	100	**100**	100	100
SG+D2nd	100	100	100	100	100	**100**	100	100	100	100
SNV+Detrend	100	100	100	100	100	99.26	100	100	100	**100**

Table S7 Precision and recall of the green mature stage category in feature wavelength modeling using SVM

**Preprocessing**	**precision (%)**					**recall (%)**				
	**SPA**	**CARS**	**IVISSA**	**CARS-SPA**	**IVISSA-SPA**	**SPA**	**CARS**	**IVISSA**	**CARS-SPA**	**IVISSA-SPA**
RAW	100	80	94.55	100	78.36	77.14	100	100	**83.49**	100
D1st	78.36	88.29	99.1	87.5	82.05	100	100	100	100	100
D2nd	85.22	90.74	90.74	88.29	91.43	100	**100**	100	100	100
SG+D1st	80	90.74	99.06	87.5	87.5	100	100	**100**	100	100
SG+D2nd	88.24	87.5	99.1	85.96	89.91	**100**	100	100	100	100
SNV+Detrend	80	82.05	94.55	88.24	78.95	100	100	100	100	**100**

Table S8 Precision and recall of the yellow mature stage category in feature wavelength modeling using SVM

**Preprocessing**	**precision (%)**					**recall (%)**				
	**SPA**	**CARS**	**IVISSA**	**CARS-SPA**	**IVISSA-SPA**	**SPA**	**CARS**	**IVISSA**	**CARS-SPA**	**IVISSA-SPA**

RAW	72.18	98.73	83.78	73.64	95.71	100	76.47	93.94	**100**	69.79
D1st	95.71	97.75	87.85	96.63	98.78	69.79	87	98.95	86	79.41
D2nd	96.51	100	98.9	96.67	100	83	**90**	90	87	91.18
SG+D1st	98.73	100	84.96	96.63	96.63	76.47	90	**98.97**	86	86
SG+D2nd	87.23	96.63	87.85	96.55	98.89	**85.42**	86	98.95	84	89
SNV+Detrend	98.73	98.78	83.78	87.23	95.777	76.47	79.41	93.94	85.42	**70.83**

Table S9 Precision and recall of the fully mature stage category in feature wavelength modeling using SVM

Preprocessing	precision (%)					recall (%)				
	SPA	CARS	IVISSA	CARS-SPA	IVISSA-SPA	SPA	CARS	IVISSA	CARS-SPA	IVISSA-SPA
RAW	100	100	100	100	100	92.53	99.44	89.53	**90.64**	98.28
D1st	100	100	100	100	100	98.28	98.87	92.35	98.31	99.44
D2nd	100	100	100	100	100	98.31	**100**	99.44	98.31	100
SG+D1st	100	100	100	100	100	99.44	100	**90.17**	98.31	98.31
SG+D2nd	100	100	100	100	100	**93.10**	98.31	92.35	98.31	99.44
SNV+Detrend	100	100	100	100	100	99.44	99.44	89.53	93.10	**98.28**

Table S10 Mean precision and recall of full wavelength classification

Preprocessing	precision (%)					recall (%)				
	ELM	KNN	RF	PLS-DA	SVM	ELM	KNN	RF	PLS-DA	SVM
RAW	91.38	91.14	89.34	76.73	94.04	89.56	86.87	89.47	76.02	92
SG	94.27	88.43	90.87	87.21	94.04	90.63	84.12	90.78	88.33	92
D1st	96.99	90.27	90.26	88.53	97.09	96.67	87.25	92.24	88.21	97.8
D2nd	96	92.01	92.48	85.85	97.39	95	90.25	92.59	86.58	98.11
SNV	92.33	90.64	88.38	90.02	95.22	89.55	86.06	88.24	89.72	94.03
Detrend	91	90.05	91.7	84.72	95.22	89	87.13	91.7	85.16	94.03
SG+D1st	96.72	90.51	92.29	88.52	97.09	96.9	87.65	92.22	88.21	97.8
SG+D2nd	97.19	90.41	92.44	87.21	98.03	96.3	87.45	92.45	86.29	96.33
SNV+Detrend	97	90.51	92.95	84.72	95.22	95	87.65	92.63	85.16	94.03

Table S11 Mean precision and recall of feature wavelength classification using ELM

Preprocessing	precision (%)					recall (%)				
	SPA	CARS	IVISSA	CARS-SPA	IVISSA-SPA	SPA	CARS	IVISSA	CARS-SPA	IVISSA-SPA
RAW	88.25	89.58	89.99	87.96	89.89	89.17	90.54	91.1	**89.75**	90.65
D1st	91.13	93.53	93.73	93.79	94.13	91.08	93.53	93.63	93.51	94.07
D2nd	91.81	97.16	95.01	91.84	96	91.79	**97.18**	94.66	92.06	95.82
SG+D1st	90.7	95.56	95.55	92.47	90.81	90.71	95.59	**95.56**	92.47	91.01
SG+D2nd	89.56	95.15	95.15	88.97	94.53	**89.44**	95.17	95.17	88.74	94.68
SNV+Detrend	91.23	91.46	90.71	90.14	93.03	91.22	91.46	90.74	90.14	**92.78**

Table S12 Mean precision and recall of feature wavelength classification using SVM

Preprocessing	precision (%)					recall (%)				
	SPA	CARS	IVISSA	CARS-SPA	IVISSA-SPA	SPA	CARS	IVISSA	CARS-SPA	IVISSA-SPA
RAW	90.73	92.91	92.78	91.21	91.36	89.89	91.97	94.49	**91.38**	89.36
D1st	91.36	95.35	95.65	94.71	93.61	89.36	95.29	97.1	94.77	92.95
D2nd	93.91	96.91	96.55	94.98	97.17	93.77	**96.67**	96.48	95.1	97.06
SG+D1st	92.91	96.91	94.67	94.71	94.71	91.97	96.67	**96.38**	94.77	94.77
SG+D2nd	91.82	94.71	95.65	94.17	96.27	**92.84**	94.77	97.1	94.1	96.15
SNV+Detrend	92.91	93.61	92.78	91.82	91.57	91.97	92.95	94.49	92.84	**89.7**
